# Feed plants, ethnoveterinary medicine, and biocultural values: insights on the Luchuan pig from Hakka communities in China

**DOI:** 10.1186/s13002-023-00613-4

**Published:** 2023-09-15

**Authors:** Yongqing Liufu, Jilong Zhou, Qiongyao Fu, Min Shao, Yaozhang Xie, Binsheng Luo

**Affiliations:** 1 Natural History Museum of Guangxi, Nanning, 530012 China; 2 Luchuan Livestock Station, Luchuan, 537700 China; 3 Lushan Botanical Garden, Jiangxi Province and Chinese Academy of Sciences, Lushan, 332900 China

**Keywords:** Luchuan pig, Local breed, Feed plants, Veterinary plants, Genetic breeding, Traditional knowledge, Hakka

## Abstract

**Background:**

The Luchuan pig is an indigenous breed from Luchuan County, China, with cultural and genetic significance. However, traditional knowledge and conservation status have not been systematically documented.

**Methods:**

Using ethnobiological methods, we surveyed 72 Luchuan pig farmers in 7 townships during 2021–2023. Semi-structured interviews and participant observation were conducted to document traditional knowledge and management practices.

**Results:**

The locals reported 51 plant species used as pig feed, with 30 wild species. Growth-stage-specific feeding and seasonal adjustment practices were documented. We recorded 62 ethnoveterinary plant uses, mainly for treating pigs' heat stress and skin conditions. Luchuan pigs play central roles in local Hakka customs, rituals, and cuisine. Additonally, the new ecological farming models minimize the environmental impacts to the local community. However, there are still some challenges remained for conserving and promoting Luchuan pigs.

**Conclusions:**

The Luchuan Hakka people possess rich traditional knowledge and management experience in raising Luchuan pigs. Our study provides extensive documentation of traditional knowledge and recommends integrating cultural and genetic aspects for sustaining this biocultural heritage. Findings can inform initiatives supporting local breed conservation globally.

**Supplementary Information:**

The online version contains supplementary material available at 10.1186/s13002-023-00613-4.

## Introduction

Livestock and poultry genetic resources provide quality animal products and promote farmer income, sustainable resource use, and ecological and cultural heritage protection, establishing a foundation for food security, rural revitalization, biodiversity conservation, and ecological construction [1–3]. Native breeds of livestock and poultry are populations adapted to local traditional farming systems and environments [4]. Originating from specific regions where they are frequently utilized, native breeds are adapted to the local conditions [4]. They often have advantageous traits like coarse feed tolerance, disease resistance, delicious taste, good meat quality, and stable genetics, making them important genetic resources for developing new breeds and promoting sustainable animal husbandry [5–7].

As a major livestock producer, China possesses the world's richest livestock and poultry genetic resources, accounting for about 1/6 of the global total [8, 9]. However, the rise of intensive farming and economic growth has led to the replacement of some native breeds with faster-growing varieties, driving rapid decline and even extinction of local breeds [10–12]. Statistics indicate that 30% of existing animal genetic resources are threatened, endangered, vulnerable, or extinct [13]. Alarmingly, nearly 800 livestock breeds have been documented as lost in the past century [13]. Surveys reveal that 55 local Chinese breeds are endangered and 22 critically endangered [14]. Additionally, 15 documented breeds were not found, making their extinction status unclear [14]. In total, endangered and critically endangered breeds account for approximately 14% of local breeds in China [14]. Thus, the attention and protection of traditional animal breeds in China is imperative.

Luchuan County in Guangxi is a major settlement area for the Hakka people, with approximately 700,000 residents, constituting about two-thirds of the total population [15]. The Luchuan pig, named after its place of origin, is primarily raised by Hakka people in the region and has become a representative indigenous breed in China. Compared to Western domestic pigs, Luchuan pigs exhibit distinct characteristics, including superior meat quality, early maturity, high reproductive capacity, stable maternal lineage, adaptability to coarse feed, and strong disease resistance [16–18]. Recognized as one of China's excellent local pig breeds, the Luchuan pig was listed in the “Catalogue of National Livestock and Poultry Genetic Resources” and “Geographical Indication Protection Products for Agricultural Products” [19]. Studies report that Luchuan pig meat is tender, delicious, and rich in essential amino acids like glutamic acid and lysine, as well as vitamins, saturated fatty acids, and monounsaturated fatty acids, contributing to its unique flavor and high nutritional value [20–22]. However, Luchuan pigs also exhibit some physiological limitations, such as small body size, slow growth rate, and low lean meat percentage [23]. Moreover, their higher market price compared to other pig breeds has led to limited market capacity. These factors pose challenges to the reproductive production of Luchuan pigs. Despite being recognized as an important genetic breeding strategic resource, the conservation of Luchuan pigs remains a complex task, drawing significant attention from the local government.

Researches have shown that the conservation of genetic resources cannot be dissociated from their natural and cultural environments [11, 24]. In numerous indigenous regions around the world, the utilization of natural resources is embedded within local traditional knowledge and culture. Traditional knowledge serves as a valuable source of information concerning local wild forage resources, their nutritional characteristics, as well as veterinary and plant resources [25]. It can significantly contribute to the development of novel and sustainable approaches to natural resource management [26, 27].

The Hakka people in Luchuan have accumulated a vast amount of traditional knowledge regarding breeding Luchuan pigs through their long-standing production practices. Alongside this knowledge, they have also developed various traditional cultures and customs related to pigs, such as dietary customs, social rituals, and festive traditions. Despite the crucial significance of this knowledge in the conservation of Luchuan pigs, there is a lack of systematic documentation and record-keeping of these traditional practices. Therefore, we conducted multiple investigations in Luchuan County with the objective of (1) documenting the traditional breeding and management experiences of Luchuan pigs, including the utilization of feed and veterinary medicinal plants; (2) evaluating and identifying key plant species used in feeding Luchuan pigs through a scoring system for feed plants; (3) assessing the current status of the local Luchuan pig-related industry's conservation and development and providing feasible improvement suggestions; and (4) elucidating the importance of Luchuan pig-related traditional customs and culture in the conservation efforts. This investigation aims to provide insights into the development of plant-based feed and veterinary medicine and also serve as a case reference for the conservation and industrial development of local livestock and poultry breeds.

## Study area and methodologies

Luchuan County, Guangxi Autonomous Region, is situated in the hilly region of south China and represents a typical agricultural area characterized by hills. The land in this region is fertile, with a substantial organic matter content (3.21%) and a considerable presence of iron elements, and the pH level ranges from 5.4 to 6.7. The climate is mild, with abundant sunlight, plentiful rainfall, and a long frost-free period, providing highly favorable conditions for the growth and reproduction of various flora and fauna. These conditions also benefit the cultivation and propagation of agricultural and forage crops, which offer an excellent natural geographical environment for breeding and raising Luchuan pigs [28].

**Fig. 1:**
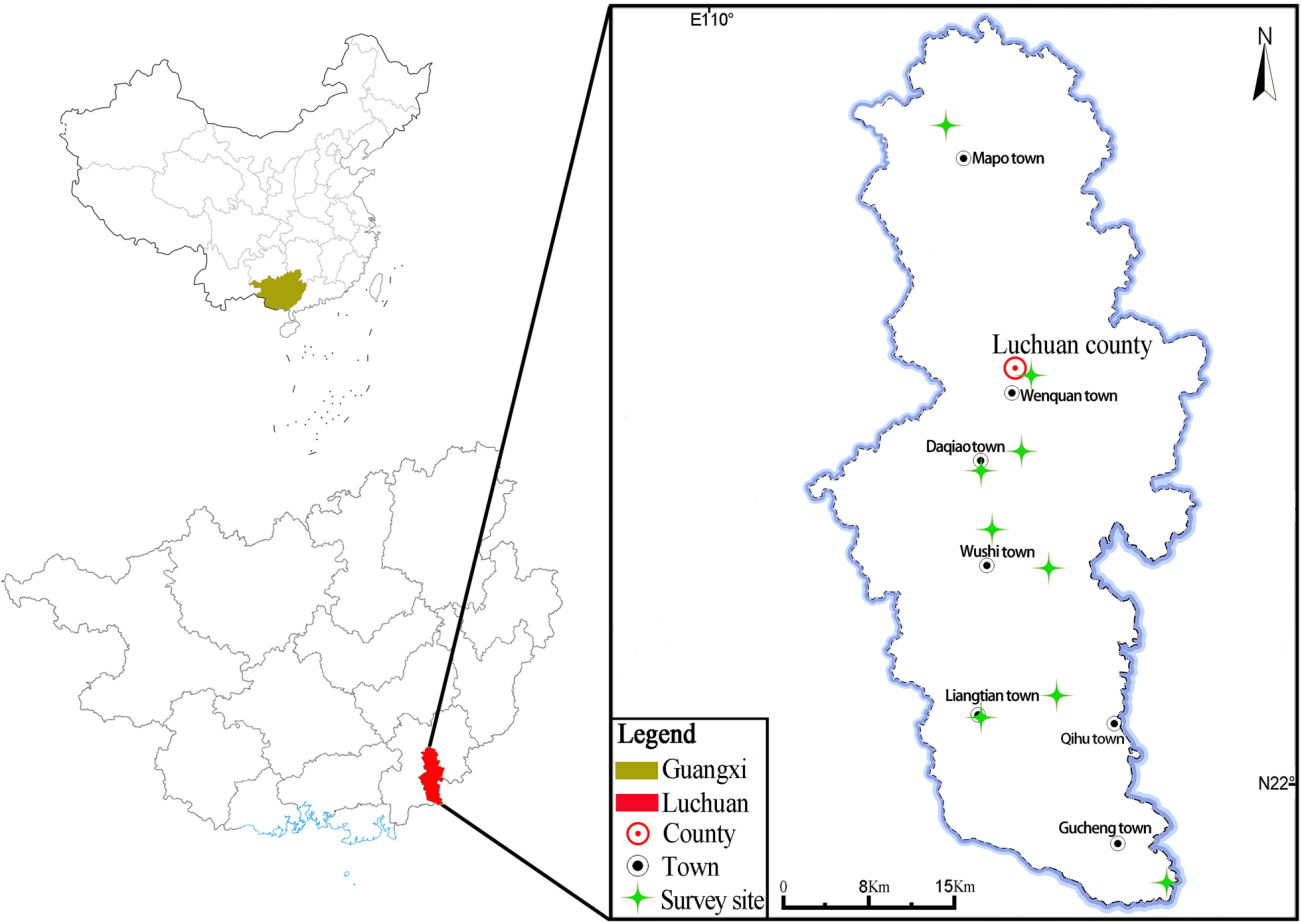
Map of the study area

From 2021 to 2023, we conducted ethnobiological research using the ethnobotanical approach in seven townships known for their significant Luchuan pig farming activities, including Gucheng, Qinghu, Liangtian, Wushi, Daqiao, Wenquan, and Mapo (Fig. 1).

Subsequently, purposive sampling was employed to select respondents with abundant traditional knowledge of pig farming for interviews [3, 29]. A total of 72 respondents participated in the study, comprising 25 females and 47 males, with an average age of 55.8 years and an average pig farming experience of 26.9 years.

Data collection employed a semi-structured interview method and participatory observation [3, 24, 30]. During the semi-structured interviews, respondents were asked basically around the questions listed in annex (Additional file 1). The participatory observation was conducted during the free-range grazing of Luchuan pigs and the collection of feed and veterinary medicinal plants by farmers. Specimens were collected, and records were made of the plant species and related knowledge on their utilization. Plant identification was carried out using taxonomic electronic databases, such as https://www.cvh.ac.cn/; http://www.iplant.cn/; www.worldfloraonline.org, based on morphological characteristics and geographical origins of the plants. Voucher specimens of all the feed plants and veterinary medicinal plants were collected and deposited in the herbarium of the Natural History Museum of Guangxi.

Furthermore, 2–3 individuals, totaling 25 key informants, including farmers, farm caretakers, veterinarians, and intangible cultural heritage inheritors, were selected at each survey site for group discussions and scoring [31]. Five aspects were quantitatively scored for the feed plants: nutritional value, pig preference level, availability of resources, digestibility, and frequency of use. Each aspect was rated on a scale from 1 to 5, with 5 indicating the highest score and 1 the lowest. For instance, regarding the nutritional value of feed plants, the scoring criteria were as follows: Excellent (5 points), Good (4 points), Moderately Good (3 points), Fair (2 points), and Poor (1 point) [30].The scoring criteria for the frequency of use were as follows: used more than once per week (5 points), used once per week (4 points), used once per month (3 points), used less than once per month but more than once per year (2 points), and used once or less per year (1 point). The overall utilization value of feed plants was assessed based on the total scores. A higher score indicated a higher comprehensive utilization value of the feed plant, indicating greater development prospects.

## Results and discussion

### The traditional feed of Luchuan pigs

**Fig. 2:**
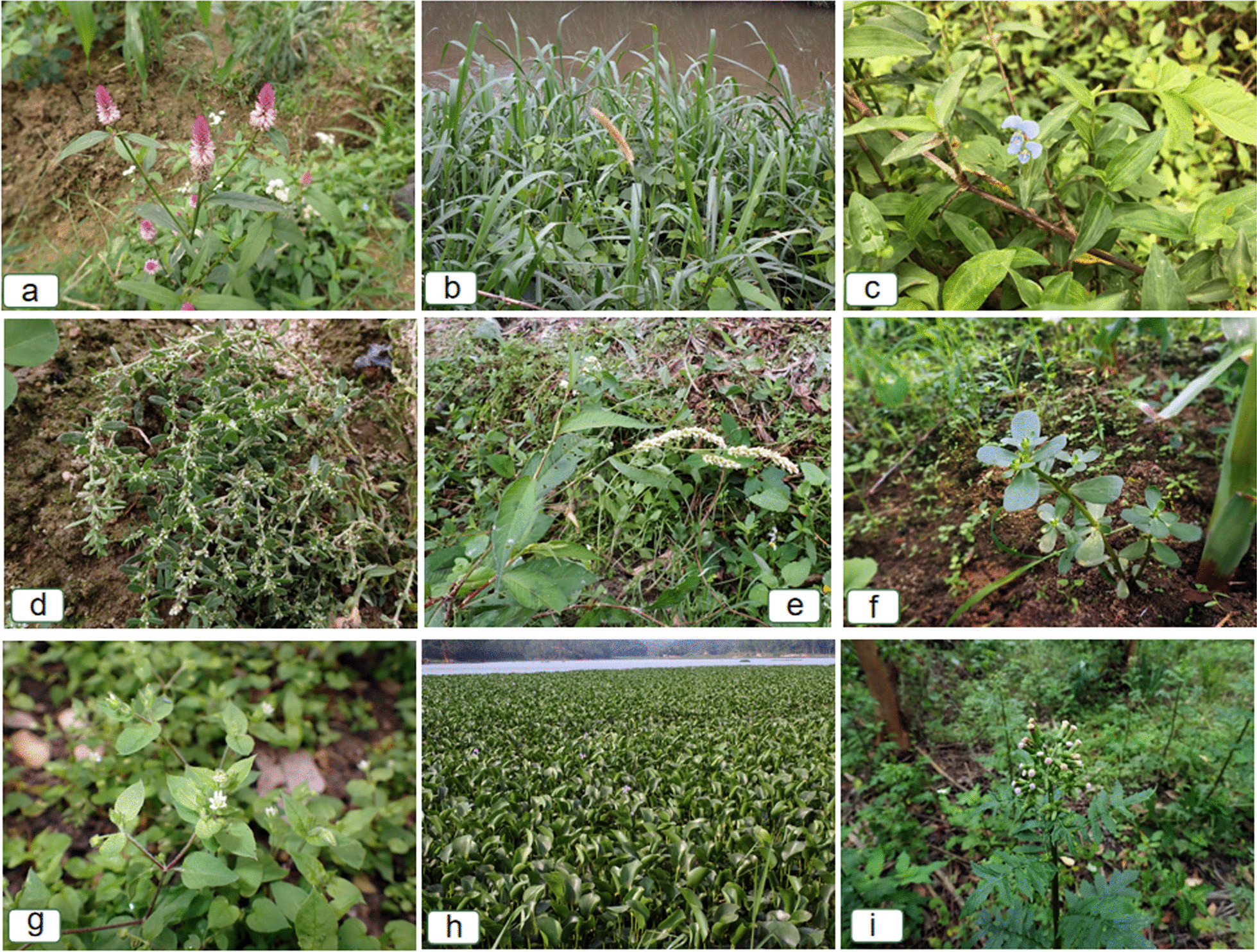
Part of wild feed plants for Luchuan Pigs. (**a** *Celosia argentea*, **b** *Pennisetum purpureum*, **c** *Commelina diffusa*, **d** *Polygonum plebeium*, **e** *Persicaria lapathifolia*, **f** *Portulaca oleracea*, **g** *Stellaria aquatica*, **h** *Pontederia crassipes*, **i** *Erechtites valerianifolius*, all photographs were taken by Yongqing Liufu.)

A total of 51 feed plants were reported by the respondents, belonging to 21 families and 43 genera (Table 1). Among these feed plants, the Asteraceae, Poaceae, and Amaranthaceae families were the most represented, with 11, 6, and 5 species, respectively. Various parts of the plants were utilized as feed, including above-ground parts, whole plants, leaves, fruits, seeds, seed coats, rice husks, stems, and root blocks, with above-ground parts and whole plants being the primary components, accounting for 37.7% and 22.6%, respectively. More than half of the feed plants used for Luchuan pigs were wild plants (accounting for 56.6% of the total) (some images are shown in Fig. 2), while cultivated plants accounted for 41.5%. The results showed that the comprehensive utilization scores of Luchuan pig feed plants ranged from 10 to 24 points. Rice (*Oryza sativa*), corn (*Zea mays)*, *Pennisetum purpureum*, sweet potato (*Ipomoea batatas*), wheat (*Triticum aestivum* subsp. *spelta*), and soybean obtained (*Glycine max*) relatively high scores, indicating their significant comprehensive utilization value in Luchuan pig farming. Apart from wheat, which needs to be introduced, all these plants are commonly cultivated locally. *P. purpureum*, on the other hand, is both harvested from the wild and cultivated by some individuals.

**Table 1:** The inventory of feed plants for Luchuan pigs

Specimen No	Family name	Scientific name	Local name	Used part	Harvesting season	Resource type	Life form	Integrated score
LFYQ23146	Amaranthaceae	*Beta vulgaris* L.	tóng sháo mài, zhū má cài	Leaf	Spring, summer, winter	Cultivated	Herb	20
LFYQ23157	Amaranthaceae	*Alternanthera philoxeroides* (Mart.) Griseb.	jiǎ kōng xīn cài	Overground part	Spring, summer	Wild	Herb	16
LFYQ23164	Amaranthaceae	*Amaranthus viridis* L.	gǒu sè xiàn	Whole plant	Spring, summer, fall	Wild	Herb	16
LFYQ23034	Amaranthaceae	*Cyathula prostrata* (L.) Blume	xì yàng mǎ biān cǎo, dì dān	Whole plant	Whole year	Wild	Herb	12
LFYQ23019	Amaranthaceae	*Celosia argentea* L.	qīng xiāng	Overground part	Spring, summer, fall	Wild	Herb	11
LFYQ23144	Araceae	*Colocasia esculenta* (L.) Schott.	yù tóu miáo, yǎng yù	Petiole	Whole year	Cultivated or wild	Herb	21
LFYQ23068	Asteraceae	*Ixeris polycephala* Cass. ex. DC.	mài cài	Leaf	Spring, winter	Cultivated	Herb	21
LFYQ23154	Asteraceae	*Lactuca sativa* L.	yóu mǎi cài	Leaf	Whole year	Cultivated	Herb	17
LFYQ23159	Asteraceae	*Lactuca sativa* var. *ramosa* Hort.	shēng cài	Leaf	Spring, winter	Cultivated	Herb	16
LFYQ22018	Asteraceae	*Emilia sonchifolia* (L.) DC.	yī diǎn hóng	Whole plant	Whole year	Wild	Herb	16
LFYQ23161	Asteraceae	*Lactuca sativa* var. *angustata* Irish ex Bremer	wō sǔn	Leaf	Spring, winter	Cultivated	Herb	15
LFYQ23111	Asteraceae	*Erechtites valerianifolius* (Link ex Spreng.) DC.	guò cháo cài	Overground part	Whole year	Wild	Herb	15
LFYQ23048	Asteraceae	*Gamochaeta pensylvanica* (Willd.) Cabrera	é ài	Whole plant	Whole year	Wild	Herb	15
LFYQ22009	Asteraceae	*Bidens pilosa* L.	yī bāo zhēn	Overground part	Whole year	Wild	Herb	14
LFYQ23054	Asteraceae	*Crassocephalum crepidioides* S. Moore	guò cháo cài, gé mìng cài	Overground part	Spring, summer, fall	Wild	Herb	14
LFYQ22172	Asteraceae	*Eclipta prostrata* (L.) L.	mò cǎo	Overground part	Whole year	Wild	Herb	12
LFYQ23164	Asteraceae	*Erigeron canadensis* L.	xiǎo péng cǎo	Overground part	Whole year	Wild	Herb	10
LFYQ23150	Brassicaceae	*Raphanus raphanistrum* subsp. *sativus* (L.) Domin	luó bo	Whole plant	Winter	Cultivated	Herb	18
LFYQ23151	Brassicaceae	*Brassica rapa* var. *glabra* Regel	huǒ tǒng cài, bái cài	Leaf	Winter	Cultivated	Herb	18
LFYQ23155	Brassicaceae	*Brassica oleracea* var. *botrytis* L.	yē zǐ cài	Overground part	Spring, winter	Cultivated	Herb	17
LFYQ23156	Brassicaceae	*Brassica oleracea* L.	bāo cài	Overground part	Spring, winter	Cultivated	Herb	17
LFYQ23160	Caricaceae	*Carica papaya* L.	mù dōng guā	Fruit	Fall	Cultivated	Tree	15
LFYQ23020	Caryophyllaceae	*Stellaria aquatica* Scop.	má sī cǎo, é cháng cài	Overground part	Whole year	Wild	Herb	12
LFYQ23151	Commelinaceae	*Commelina diffusa* Burm.f.	ròu cǎo, zhú gāo cǎo	Whole plant	Whole year	Wild	Herb	18
LFYQ23142	Convolvulaceae	*Ipomoea batatas* (L.) Lam.	hóng shǔ téng, hóng shǔ téng, fān shǔ	Whole plant	Whole year	Cultivated	Herb	24
LFYQ23158	Convolvulaceae	*Ipomoea aquatica* Forssk.	kōng xīn cài	Overground part	Summer	Cultivated	Herb	16
LFYQ23100	Costaceae	*Hellenia speciosa* (J.Koenig) Govaerts	fú shǒu gùn	Overground part	Spring, summer, fall	Wild	Herb	11
LFYQ23148	Cucurbitaceae	*Cucurbita moschata* Duchesne	nán guā	Fruit	Summer, fall	Cultivated	Herb	19
LFYQ23153	Cucurbitaceae	*Benincasa hispida* Cogn.	dōng guā	Fruit	Summer, fall	Cultivated	Herb	18
LFYQ23145	Euphorbiaceae	*Manihot esculenta* var. *Pohlii* Cif.	mù shǔ	Tuber	Fall, winter	Cultivated	Shrub	21
LFYQ23143	Fabaceae	*Glycine max* (L.) Merr.	dòu pò	Seed	Fall	Cultivated	Herb	23
LFYQ23147	Fabaceae	*Arachis hypogaea* L.	huā shēng fū	Seed coat	Summer	Cultivated	Herb	20
LFYQ23149	Moraceae	*Broussonetia papyrifera* (L.) Vent.	gòu shù	Tender leaf	Whole year	Wild	Tree	19
LFYQ23148	Onagraceae	*Ludwigia adscendens* (L.) H. Hara	guò táng shé	Whole plant	Whole year	Wild	Herb	19
LFYQ23021	Onagraceae	*Ludwigia hyssopifolia* (G. Don) Exell.	/	Overground part	Spring, winter	Wild	Herb	12
LFYQ22784	Poaceae	*Zea mays* L.	yù mǐ	Seed, stem	Fall	Cultivated	Herb	24
LFYQ22808	Poaceae	*Oryza sativa* L.	zhōu, mǐ kāng, xǐ mǐ shuǐ	Seed, husk	Summer, fall	Cultivated	Herb	24
LFYQ23052	Poaceae	*Pennisetum purpureum* Schumach.	tián xiàng cǎo, jiǎ gān zhè	Overground part	Whole year	Cultivated or wild	Herb	24
LFYQ22806	Poaceae	*Triticum aestivum* subsp. *spelta* (L.) Thell.	mài pí	Seed coat	/	Introduced	Herb	23
LFYQ23152	Poaceae	*Cenchrus flaccidus* (Griseb.) Morrone	huáng zhú cǎo	Overground part	Whole year	Wild	Herb	18
LFYQ23018	Poaceae	*Eleusine indica* Gaertn.	niú jīn cǎo	Overground part	Whole year	Wild	Herb	13
LFYQ23050	Polygonaceae	*Rumex crispus* L.	jiǎ mài cài	Overground part	Spring, summer, fall	Wild	Herb	16
LFYQ23015	Polygonaceae	*Persicaria maculosa* Gray	xiǎo là liǎo	Whole plant	Whole year	Wild	Herb	15
LFYQ23006	Polygonaceae	*Persicaria lapathifolia* (L.) Delarbre	jiǎ là liǎo	Overground part	Whole year	Wild	Herb	15
LFYQ23002	Polygonaceae	*Polygonum plebeium* R. Br.	wū yíng yì, páng xiè yǎn	Whole plant	Whole year	Wild	Herb	13
LFYQ23152	Pontederiaceae	*Pontederia crassipes* Mart.	shuǐ piāo, fú shuǐ lián	Overground part	Spring, summer	Wild	Herb	18
LFYQ23066	Portulacaceae	*Portulaca oleracea* L.	mǎ chǐ xiàn	Whole plant	Spring, summer, fall	Wild	Herb	16
LFYQ23163	Sapindaceae	*Litchi chinensis* Sonn.	lì zhī	Leaf	Whole year	Cultivated	Tree	10
LFYQ23013	Saururaceae	*Houttuynia cordata* Thunb.	yú xīng cǎo	Whole plant	Whole year	Wild	Herb	16
LFYQ23157	Solanaceae	*Solanum americanum* Mill.	bái huā cài	Stem and leaf	Spring, summer, fall	Wild	Herb	17
LFYQ23162	Solanaceae	*Physalis angulata* L.	dēng lóng cài	Overground part	Whole year	Wild	Herb	12

This study shows that Luchuan pig feed plants are mainly herbaceous plants (accounting for 92.2%). This result is similar to other ethnobotanical cases in Europe and other regions of Guangxi [30, 32]. The wild herbaceous plants we recorded are mainly weeds, among which *Pennisetum purpureum*, *Colocasia esculenta*, *Commelina diffusa*, *Pontederia crassipes*, *Solanum americanum*, *Emilia sonchifolia*, *Portulaca oleracea* are the most favorite forage plants harvested by local Hakka people. On the one hand, these plants are commonly found in the wastelands and ditches near their homes, which provide a convenient source of feed during periods of feed shortage. On the other hand, for local Hakka people, these plants are well-known and have multiple uses: *S. americanum*, *E. sonchifolia*, and *P. oleracea* are commonly used as wild vegetables and medicinal plants. *P. crassipes* is a commonly used weaving plant by Hakka people. *P. purpureum* and *C. diffusa* serve as fodder plants for local cattle and other livestock. The leafstalk of *C. esculenta* is utilized by locals to make pickled food, while its root is a commonly used starchy edible plant.

Among the 51 feed plants, the farmers classified rice, corn, soybean, peanut bran, and wheat, which have relatively high nutritional content, as concentrate feed. The remaining 46 feed plants, including *P. purpureum*, sweet potato (*I. batatas*), and taro (*C. esculenta*), were categorized as roughage. Different species of concentrate and roughage supplements are provided to Luchuan pigs during different growth stages. During the gestation period, it is essential to balance the nutritional needs of pregnant sows. For piglets aged 0 to 2 months, their protein requirements are high, and they primarily rely on maternal milk supplemented with appropriate solid food. They should be fed 4 to 5 times a day with a diet consisting mainly of porridge, corn (*Z. mays*) flour, soybean (*G. max*) meal, and wheat (*T. aestivum* subsp*.* *spelta*) bran, mixed with small amounts of fish meal and bone meal. After reaching a weight of 7.5 kg, roughage feed should be gradually introduced. Piglets can be kept in confinement after weaning or gradually switched to free-range feeding. When confined, they should be fed twice a day, with roughage as the main component and a small amount of concentrate feed. During free-range feeding, they can be fed once in the morning or evening, with the rest of the time spent foraging on wild plants.

The favorable weather and unique geographical environment have nurtured abundant feed plant resources in Luchuan. The survey results reveal that a large variety of plants are available year-round to feed Luchuan pigs. Among the feed plants used for Luchuan pigs, 23 species can be harvested throughout the year, accounting for 45.10% of the total; 16 species are harvested in spring (31.37%), 14 species in summer (27.45%), 13 species in autumn (24.49%), and 10 species in winter (19.61%). During late winter and early spring, farmers mainly compensate for the insufficient feed by strengthening the cultivation of melons, fruits, and vegetables. Some farmers also utilize fermentation techniques to preserve plant feed, such as fermenting *P. purpureum*. They rarely use artificial feed, as they believe that feeding artificial feed may lead to issues such as fever, constipation, and digestive problems in Luchuan pigs.

During our participatory survey of free-range pigs, we found that Luchuan pigs consume a greater variety of wild plants than that the respondents reported, such as *Eleusine indica*, *Ludwigia adscendens*, *Erigeron canadensis*. The respondents who raised free-range pigs mentioned that they were familiar with certain plants but did not know their specific names. However, when we interviewed the handlers of captive-bred Luchuan pigs, they were able to name the commonly used feed plants and their other functions. A study from Nigeria also found that farmers who kept confined animals had a greater knowledge about the resources compared with farmers who raised animals free in the pasture [33].

Compared to the present, in the past, farmers used more wild plants to feed Luchuan pigs, especially during times of grain scarcity when people had no choice but to rely on wild plants. However, with improvements in living standards and changes in feeding practices, many traditional wild-feed plants have been replaced by fermented feed and cultivated plants. Similar to other Chinese local pig breeds [29], the traditional knowledge about the utilization and management of wild-feed plants is facing significant risks of extinction in the local community.

### The traditional veterinary medicine for Luchuan pigs

**Fig. 3:**
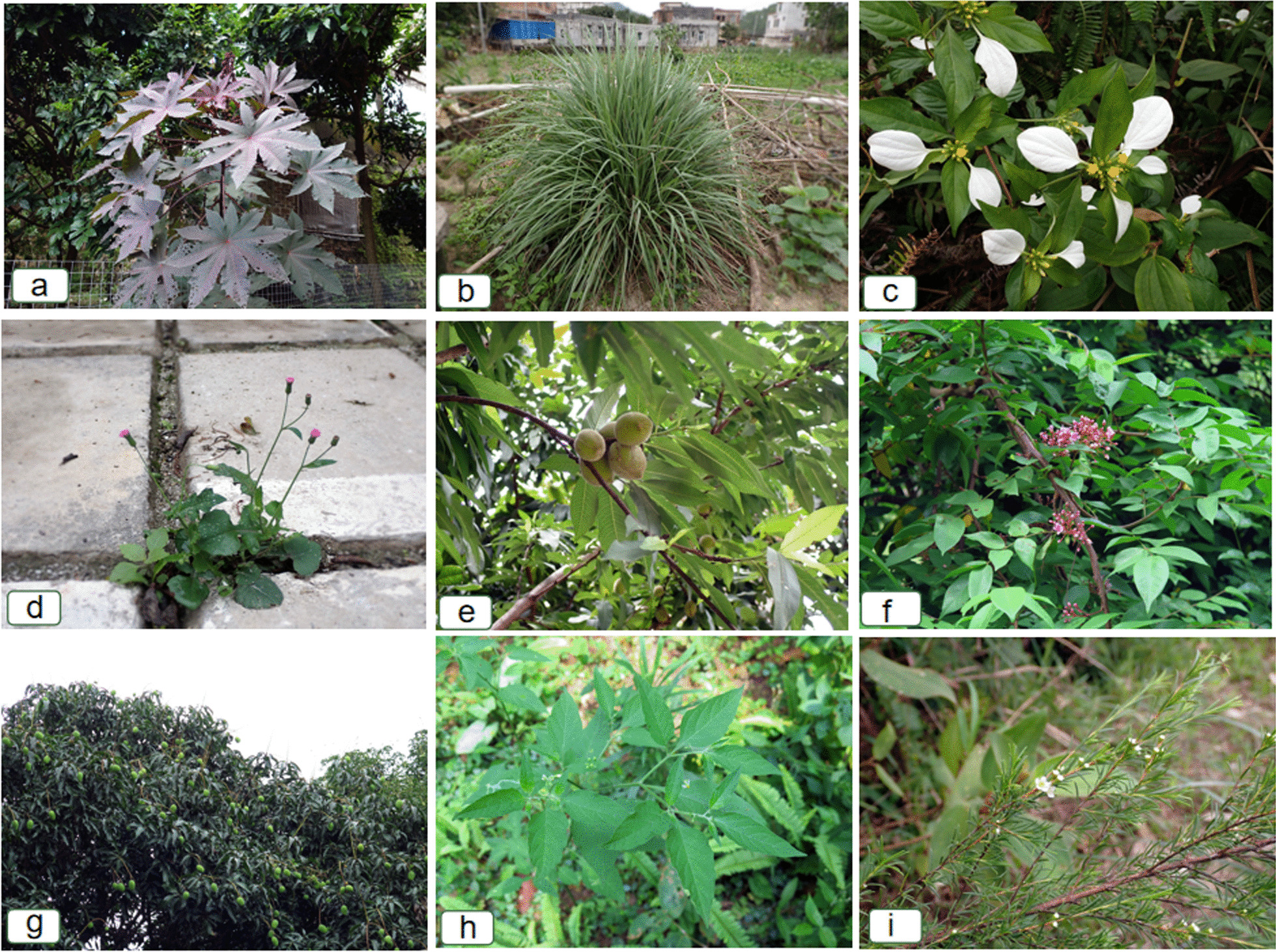
Part of veterinary plants for Luchuan Pigs. (**a** *Ricinus communis*,** b** *Cymbopogon citratus*,** c** *Mussaenda pubescens*, **d** *Emilia sonchifolia*, **e** *Prunus persica*, **f** *Averrhoa carambola*, **g** *Mangifera indica*, **h** *Solanum americanum*, **i** *Baeckea frutescen*s, all photographs were taken by Yongqing Liufu.)

Traditional herbal medicine is an important source of medication used by farmers to treat ailments in Luchuan pigs. The results of this study show that a total of 62 species of herbal medicines were reported by the respondents for treating diseases in Luchuan pigs (Table 2**, **Fig. 3). Common diseases in Luchuan pigs include cold, cough, wheezing, constipation, anorexia, indigestion, internal heat, and diarrhea.

**Table 2:** The inventory of veterinary plant for Luchuan Pigs

Specimen ID	Family name	Scientific name	Local name	Part used	Processing method	Medicinal effect	Life form	Resource type
LYFQ21020	Acanthaceae	*Dicliptera chinensis* Juss.	qīng shé	Overground part	Cook fully and feed	Clear heat, treat cold, treat jaundice	Herb	Wild
LFYQ23170	Acanthaceae	*Strobilanthes cusia* Kuntze	bǎn lán gēn	Root	Make herbal decoction and drink; sun-dry, grind into powder, and mix into feed	Clear heat, treat jaundice	Herb	Introduced
LFYQ23175	Acanthaceae	*Andrographis paniculata* (Burm.f.) Wall.	chuān xīn lián	Overground part	Sun-dry, grind into powder, and mix into feed	Clear heat, cool blood, reduce swelling, treat cold, reduce fever	Herb	Introduced
LYFQ21121	Anacardiaceae	*Mangifera indica* L.	máng guǒ mù yè	Leaf	Make herbal decoction and drink	Improve digestion	Tree	Cultivated
LYFQ21027	Apiaceae	*Hydrocotyle sibthorpioides* Lam.	xì yàng léi gōng gēn	Whole plant	Cook fully and feed	Treat cold, treat cough	Herb	Wild
LFYQ23177	Apiaceae	*Saposhnikovia divaricata* (Turcz.) Schischk.	fáng fēng	Root	Sun-dry, grind into powder, and mix into feed	Treat cold	Herb	Introduced
LYFQ21007	Apiaceae	*Centella asiatica* (L.) Urb.	yú sè cǎo	Whole plant	Cook fully and feed	Treat jaundice, clear heat	Herb	Wild
LFYQ22348	Aquifoliaceae	*Ilex rotunda* Thunb.	róng dǎn mù	Root	Make herbal decoction and drink	Clear heat, treat cold	Tree	Wild
LFYQ22021	Asteraceae	*Elephantopus scaber* L.	dì dǎn tóu	Whole plant	Make herbal decoction and drink	Clear heat, treat cold, treat jaundice	Herb	Wild
LYFQ21157	Asteraceae	*Ageratum conyzoides* L.	chòu cǎo	Overground part	Make herbal decoction for bath	Kill bacterial, relieve itching, treat skin diseases	Herb	Wild
LYFQ21002	Asteraceae	*Emilia sonchifolia* (L.) DC.	yī diǎn hóng	Overground part	Cook fully and feed	Clear heat	Herb	Wild
LFYQ23054	Asteraceae	*Taraxacum mongolicum* Hand.-Mazz.	pú gōng yīng	Whole plant	Cook fully and feed	Relieve inner heat	Herb	Introduced
LFYQ23174	Asteraceae	*Artemisia annua* L.	qīng hāo	Overground part	Make herbal decoction and drink	Treat gastrointestinal illnesses	Herb	Introduced
LYFQ21084	Asteraceae	*Artemisia indica* Willd.	ài	Overground part	Herbal soak or decoction for bathing	Relieve itching, expel parasites, treat skin diseases	Herb	Wild
LFYQ23183	Asteraceae	*Artemisia argyi* H.Lév. & Vaniot	ài	Overground part	Herbal soak or decoction for bathing	Relieve itching, expel parasites, treat skin diseases	Herb	Wild
LYFQ21242	Caprifoliaceae	*Lonicera confusa* DC.	jīn yín huā	Branch and leaf; flower	Flower or branch and leaf: make herbal decoction and drink; branch and leaf: herbal soak or decoction for bathing	Herbal drink: treat cold, clear heat; medicinal bath: relieve itching, expel parasites, treat skin diseases	Liana	Wild
LFYQ23182	Celastraceae	*Tripterygium wilfordii* Hook. f	léi gōnɡ ténɡ	Root	Herbal soak or decoction for bathing	Relieve itching, expel parasites, treat skin diseases	Shrub	Wild
LYFQ21217	Convolvulaceae	*Cuscuta australis* R.Br.	tú sī zǐ	Whole plant	Cook fully and feed	Alleviate constipation	Herb	Wild
LYFQ21297	Dioscoreaceae	*Schizocapsa plantaginea* Hance	shuǐ tián qī	Whole plant	Make herbal decoction and drink	Clear heat, treat pink eye	Herb	Wild
LFYQ23181	Dryopteridaceae	*Dryopteris crassirhizoma* Nakai	guàn zhòng	Root	Make herbal decoction and drink	Clear heat, stimulate urination, alleviate inflammation	Herb	Introduced
LFYQ23178	Ephedraceae	*Ephedra equisetina* Bunge	má huáng	Stem	Sun-dry, grind into powder, and mix into feed	Clear heat, treat asthma, treat lung fever	Shrub	Introduced
LYFQ21159	Euphorbiaceae	*Breynia fruticosa* (L.) Müll.Arg	guǐ huà fú	Branch and leaf	Make herbal decoction and drink	Stimulate urination, treat dysentery	Shrub	Wild
LFYQ22341	Euphorbiaceae	*Ricinus communis* L.	hóng bì má	Branch and leaf	Herbal soak or decoction for bathing	Relieve itching, expel parasites, treat skin diseases	Herb	Cultivated
LYFQ21038	Fabaceae	*Grona styracifolia* (Osbeck) H. Ohashi & K. Ohashi	jīn qián cǎo	Whole plant	Make herbal decoction and drink	Improve digestion	Herb	Wild
LFYQ23171	Fabaceae	*Glycyrrhiza uralensis* Fisch.	gān cǎo	Root	Sun-dry, grind into powder, and mix into feed; make herbal decoction and drink	Treat asthma	Herb	Introduced
LFYQ23172	Fabaceae	*Astragalus mongholicus* Bunge	huáng qí	Root	Make herbal decoction and drink	Treat gastrointestinal illnesses	Herb	Introduced
LFYQ23166	Gentianaceae	*Gentiana scabra* Bunge	dǎn cǎo	Whole plant	Sun-dry, grind into powder, and mix into feed	Clear heat, treat cold	Herb	Wild
LYFQ21192	Lamiaceae	*Clerodendrum fortunatum* L.	hóng dēng lóng	Overground part	Make herbal decoction and drink	Alleviate swine fever	Herb	Wild
LYFQ21046	Lamiaceae	*Mentha canadensis* L.	bò hé	Overground part	Crush and mix into feed; cook fully and feed	Treat cold	Herb	Wild
LFYQ23176	Lamiaceae	*Schizonepeta tenuifolia* Briq.	jīng jiè	Overground part	Sun-dry, grind into powder, and mix into feed	Treat cold	Herb	Introduced
LYFQ21094	Lamiaceae	*Leonurus japonicus* Houtt.	yì mǔ cǎo	Overground part	Cook fully and feed	Prevent miscarriage	Herb	Wild
LYFQ21064	Lygodiaceae	*Lygodium japonicum* (Thunb.) Sw.	niú dòu xū	Branch and leaf	Make herbal decoction for bath; place fresh plants in pigpen as bedding	Kill bacterial	Herb	Wild
LYFQ21172	Melastomataceae	*Melastoma dodecandrum* Lour.	dì niè	Whole plant	Cook fully and feed	Stop diarrhea	Herb	Wild
LFYQ22427	Meliaceae	*Melia azedarach* L.	kǔ liàn mù	Bark	Herbal soak or decoction for bathing	Relieve itching, expel parasites, treat skin diseases	Tree	Wild
LFYQ23137	Menispermaceae	*Fibraurea recisa* Pierre	shān dà wáng, shān huáng lián	Root, stem	Make herbal decoction and drink	Clear heat	Liana	Wild
LFYQ22811	Musaceae	*Musa acuminata* Colla	jiāo xīn	Leaf sheath	Cook fully and feed	Relieve constipation, treat heat syndrome of the eyes, clear heat	Herb	Cultivated
LFYQ23036	Myrtaceae	*Psidium guajava* L.	fān táo	Tender leaf	Make herbal decoction and drink	Stop diarrhea	Tree	Cultivated
LFYQ23028	Myrtaceae	*Baeckea frutescens* L.	sào bǎ	Tender branch and leaf	Herbal soak or decoction for bathing	Relieve itching, expel parasites, treat skin diseases	Shrub	Wild
LFYQ22745	Myrtaceae	*Eucalyptus robusta* Sm.	dà yè ān	Branch and leaf	Herbal soak or decoction for bathing	Relieve itching, expel parasites, treat skin diseases	Tree	Cultivated
LFYQ23180	Oleaceae	*Forsythia suspensa* Vahl	lián qiào	Fruit	Sun-dry, grind into powder, and mix into feed	Clear heat	Shrub	Introduced
LFYQ23148	Onagraceae	*Ludwigia adscendens* (L.) H.Hara	guò táng shé	Whole plant	Cook fully and feed	Clear heat, treat cold, relieve constipation	Herb	Wild
LFYQ23167	Oxalidaceae	*Averrhoa carambola* L.	yáng táo mù yè	Branch and leaf	Make herbal decoction and drink	Improve digestion	Tree	Wild
LYFQ21143	Pandanaceae	*Pandanus tectorius* Parkinson ex Du Roi	gāo jiǎo lǜ gǔ tóu	Fruit	Make herbal decoction and drink	Clear heat	Shrub	Wild
LFYQ23095	Pinaceae	*Pinus massoniana* Lamb.	sōng zhēn	Branch and leaf	Herbal soak or decoction for bathing	Relieve itching, expel parasites, treat skin diseases	Tree	Wild
LYFQ21022	Plantaginaceae	*Plantago major* L.	zhú ké cài	Whole plant	Cook fully and feed	Stimulate urination	Herb	Wild
LYFQ21166	Poaceae	*Lophatherum gracile* Brongn.	dàn zhú yè	Whole plant	Make herbal decoction and drink	Improve digestion, clear heat, treat cold	Herb	Wild
LYFQ21278	Poaceae	*Cymbopogon citratus* Stapf	xiāng máo	Leaf	Make herbal decoction for bath; place fresh plants in pigpen as bedding	Sterilize, kill bacterial, expel evil	Herb	Wild
LFYQ23169	Poaceae	*Bambusa blumeana* Schult. f.	lè zhú xīn	Tender leaf	Make herbal decoction and drink	Clear heat	Herb	Cultivated
LYFQ21071	Poaceae	*Panicum repens* L.	yìng gǔ cǎo shǔ	Root	Make herbal decoction and drink	Alleviate stomach distension	Herb	Wild
LFYQ23173	Poaceae	*Phragmites australis* (Cav.) Steud.	wěi gēn	Root	Sun-dry, grind into powder, and mix into feed; make herbal decoction and drink	Treat gastrointestinal illnesses	Herb	Wild
LFYQ22184	Polygonaceae	*Polygonum chinense* L.	huǒ zhǐ tàn chā	Branch and leaf	Cook fully and feed; make herbal decoction for bath	Feed: clear heat, swine fever, prevent miscarriage, treat jaundice; decoction for bathing: relieve itching, treat skin diseases	Herb	Wild
LFYQ23066	Portulacaceae	*Portulaca oleracea* L.	mǎ chǐ xiàn	Whole plant	Cook fully and feed	Relieve constipation	Herb	Wild
LFYQ23165	Primulaceae	*Maesa perlarius* (Lour.)Merr.	jì yú dǎn	Branch and leaf	Make herbal decoction and drink	Clear heat, treat cold, treat jaundice	Shrub	Wild
LFYQ23083	Primulaceae	*Embelia laeta* (L.) Mez	suān téng mù yè	Leaf, root	Make herbal decoction and drink	Leaf: improve digestion, stop diarrhea, treat jaundice, clear heat; root: clear heat	Liana	Wild
LFYQ23168	Rosaceae	*Prunus persica* (L.) Stokes	máo táo yè	Branch and leaf	Make herbal decoction for bath; place fresh plants in pigpen as bedding	Sterilize, kill bacterial, expel evil	Tree	Cultivated
LFYQ23179	Rosaceae	*Prunus sibirica* L.	kǔ xìng rén	Seed	Sun-dry, grind into powder, and mix into feed	Clear heat, treat asthma, treat lung heat	Shrub	Introduced
LFYQ23123	Rubiaceae	*Psychotria serpens L.*	shàng mù shé	Whole plant	Cook fully and feed	Clear heat, treat cold	Liana	Wild
LFYQ22015	Rubiaceae	*Mussaenda pubescens* W.T.Aiton	xiǎo liáng téng	Whole plant	Make herbal decoction and drink	Treat cold, clear heat	Shrub	Wild
LYFQ21005	Saururaceae	*Houttuynia cordata* Thunb.	yú xīng cǎo	Whole plant	Cook fully and feed; or crush and mix into feed	Treat cold, treat cough	Herb	Wild
LYFQ21021	Solanaceae	*Solanum americanum* Mill.	bái huā cài	Overground part	Cook fully and feed	Clear heat, treat jaundice	Herb	Wild
LFYQ23010	Urticaceae	*Boehmeria nivea* Gaudich.	zhù má yè	Branch and leaf	Make herbal decoction and drink	Prevent miscarriage, alleviate heat syndrome in sows	Shrub	Wild
LYFQ21149	Verbenaceae	*Clerodendrum cyrtophyllum* Turcz.	dà qīng yè	Root	Make herbal decoction and drink	Antibacterial, stimulate urination, treat jaundice, clear heat	Tree	Wild

Pig farmers mentioned that feeding Luchuan pigs with rice (*O. sativa*) bran, soybean (*G. max)* meal, and peanut (*Arachis hypogaea*) bran can easily lead to internal heat and associated issues such as anorexia, constipation, and wheezing. Therefore, they place great emphasis on preventing internal heat in the process of pig farming. They use 27 species of crushed or grinded herbs decocted and mixed into the feed as a preventive and treatment measure for internal heat in Luchuan pigs. Locally, these herbal concoctions are referred to as “cooling teas”. Additionally, they adjust the daily diet of Luchuan pigs to prevent and treat internal heat. For instance, when symptoms of internal heat occur in Luchuan pigs, the feeders reduce the proportion of rice (*O. sativa*) bran, soybean (*G. max)* meal, and peanut (*Arachis hypogaea*) bran in their food while increasing the proportion of wheat (*T. aestivum* subsp. *spelta*) bran, sweet potato (*I. batatas*) shoots, and *Musa acuminata*. These plants promote gastrointestinal motility in pigs, thereby alleviating symptoms such as constipation and indigestion.

According to the interviewees, newborn piglets and pigs raised under forested conditions are prone to skin diseases. To address this, the local people use 14 species of plants, such as *Pinus massoniana*, *Melia azedarach*, *Eucalyptus robusta*, *Tripterygium wilfordii*, and *Cymbopogon citratus*, for pig bathing. They believe that these plants have excellent therapeutic effects and can treat and prevent approximately 95% of skin diseases. Research results indicate that active ingredients in plants like *E. robusta*, and *T. wilfordii* have vermifugal, insecticidal, and antibacterial effects [34–36]. In traditional Chinese medicine, *T. wilfordii*is also used for treating autoimmune diseases like rheumatoid arthritis, glomerulonephritis, and systemic lupus erythematosus [36]. During the survey, it was observed that farmers prefer placing *C. citratus*, *Prunus persica* leaves, and *Lygodium japonicum* directly inside the pigsty for pigs to sleep on. According to their accounts, this practice effectively repels mosquitoes, parasites and prevents skin diseases in piglets. *C. citratus* emits a strong aroma and is reported to have effective chemical components with antibacterial and insect-repelling properties [37–39].

It is worth noting that among the medicinal plants we have documented, three of them have been reported to posse toxicity. For example, *M. azedarach* has been reported that the bark of *M. azedarach* has cytotoxic effects and may result in gastrointestinal, cardiovascular, respiratory, or neurological effects, and death in severe cases [40, 41]. Also, the commonly used industrial plant *Ricinus communis* is also used locally; it contains highly toxic compounds such as *Ricinus communis* agglutinin and the alkaloid ricinine [42]. Besides, *Tripterygium wilfordii* has toxicity and adverse reactions, especially the hepatotoxicity [43]. These plants are mainly used by local people to relieve itching, repel insects, and treat other skin diseases in Luchuan pigs. The toxic herbal species can be potentially lethal, but their effects are closely tied to their processing methods, usage, and dosage [41, 44]. In the context of animal health, knowledge of various toxic and poisonous species is a prerequisite for safe grazing as grazing on such species could be fatal resulting in economic loss [45, 46]. Therefore, the utilization and scientific validation of these related plants should also draw the attention of local residents, local governments, and researchers.

In the past, the treatment of Luchuan pigs’ illnesses mainly relied on herbal medicine. With the development of modern medical technology, modern farmers now primarily depend on vaccines and Western medicine to treat pig diseases. Indeed, animal vaccination has significantly contributed to the prevention and control of severe animal diseases. However, this shift toward modern medical practices may have adversely affected the traditional knowledge system concerning livestock health and welfare, as vaccines have become readily available for most farmers, and disease prevention is now the primary focus [1]. Nonetheless, in treating less severe health issues and in more isolated regions, traditional ethnic veterinary practices may still serve as an essential low-cost alternative to “Western” veterinary methods [1]. Therefore, effective measures should be taken to promote the use of traditional herbal remedies. For instance, utilizing pig farming association platforms to disseminate common knowledge about traditional Chinese veterinary medicine among farmers.

### Luchuan pig farming models

**Fig. 4:**
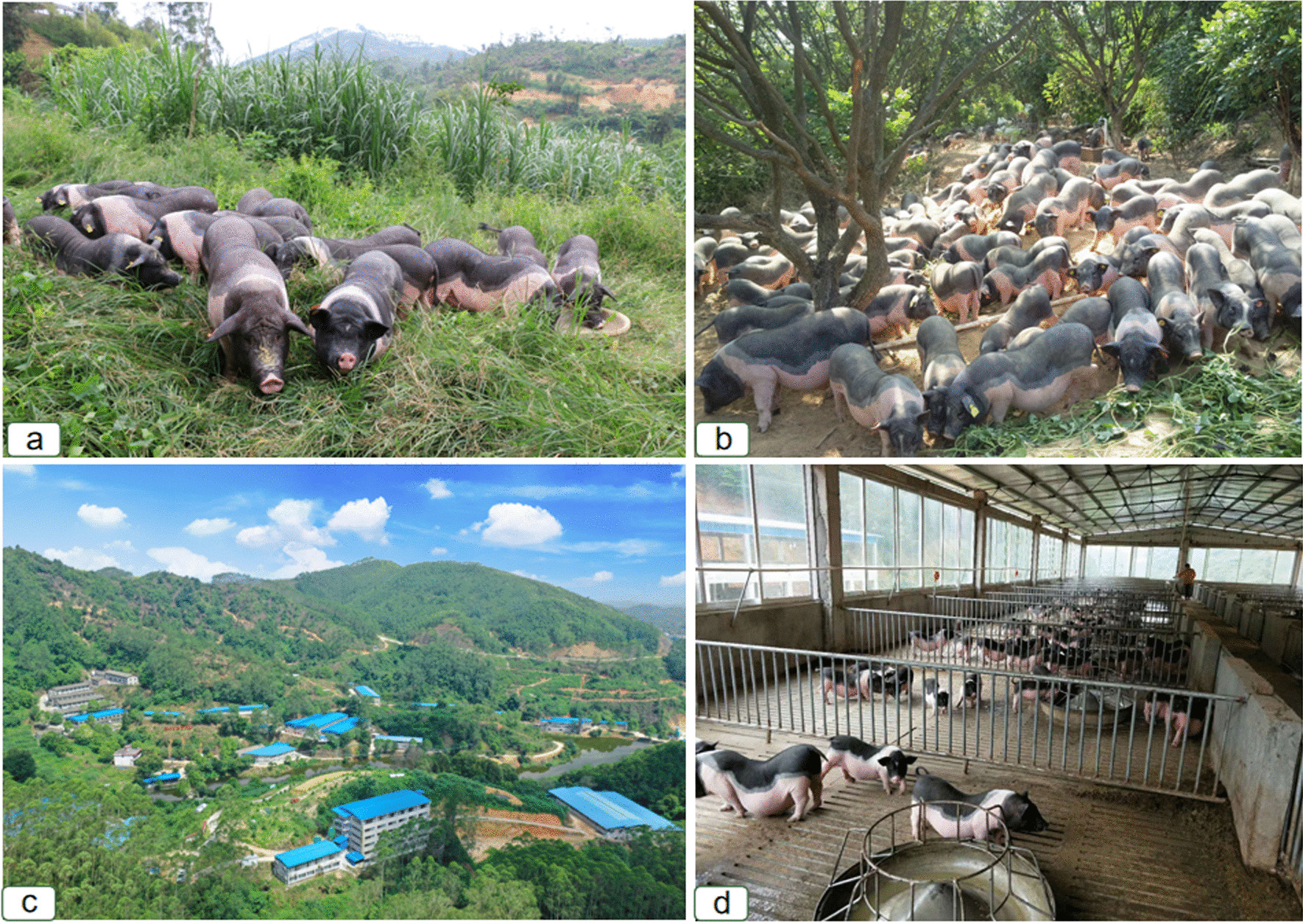
The ecological farming and free-range farming of Luchuan Pigs. (**a** free-range farming on mountain areas (by Song Li), **b** free-range farming in fruit ranch (by Guixin Chen), **c** the ecological farming base (by Jinming Li), **d** Luchuan Pigs on slatted floors (by Song Li))

In our investigation, we observed a historic transformation in the Luchuan pig farming industry, achieved through collaborative efforts among the government, enterprises, and farmers, transitioning from traditional confinement systems to more environmentally friendly and sustainable practices: ecological farming and free-range in the forest (Fig. 4).

In the ecological farming model, farms are typically established within artificial economic and fruit forests, accompanied by fish ponds. The pig pens are equipped with innovative features such as slatted floors, automated feeding and watering systems, and centralized manure treatment pools. The use of automated equipment significantly reduces feed and labor costs. Moreover, the slatted floors in the pig pens enhance ventilation, reducing the risks of harmful gases for the pigs and lowering the likelihood of diseases among Luchuan pigs. To mitigate environmental pollution from pig feces and urine, farmers primarily employ techniques such as solid–liquid separation and biogas fermentation to treat the excrement in an eco-friendly manner. The processed pig manure serves as a natural fertilizer for fruit trees, forest plants, and pasture within the farm, while the biogas slurry is used to raise fish or irrigate crops. This “pig farming + fish farming” and “pig farming + cultivation” ecological farming model not only minimizes environmental pollution from waste but also promotes sustainability and generates additional economic income for the farmers.

In the free-range in the forest model, Luchuan pig farmers divide the mountainous area into several sections and practice low-density rotational grazing to allow for free-range feeding. This method helps reduce the potential threats to the ecological environment caused by overgrazing. To manage Luchuan pigs more efficiently during the night and provide shelter during adverse weather conditions (such as rain or high temperatures), farmers often build simple houses on the mountain. Luchuan pigs in the forest are familiar with their owner’s voice or whistle and can return to designated areas based on specific signals. Under this model, Luchuan pigs are only fed once a day, which reduces feed costs. Due to the minimal input required and low economic risks, free-range pig farming is gaining popularity and plays a significant role in the livelihoods of many rural families worldwide [47–49].

In the wild, pigs are active during the day and spend 75% of their active time on foraging activities [50]. Their increased activity contributes to better health and improved meat quality compared to typical domestic pigs [3, 30, 51, 52]. Research indicates that free-range pigs living in vast, comfortable natural environments have enhanced immunity and disease resistance [3].

### Breeding utilization and industrial development of Luchuan pigs

**Fig. 5:**
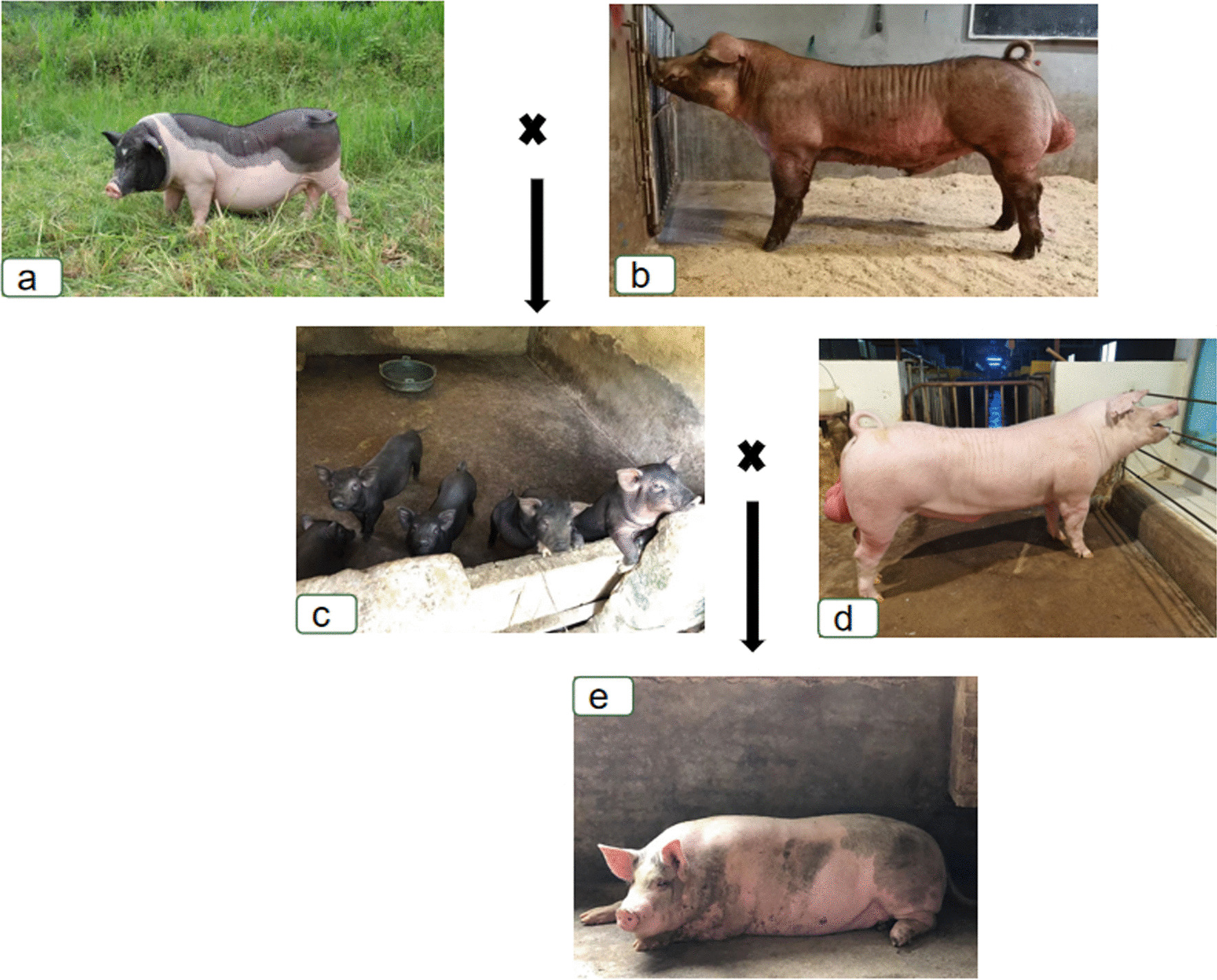
The breeding application of Luchuan Pigs. (**a** Luchuan pig (by Song Li), **b** Duroc boar (by Shichong Wang), **c** Hybrid black pig (by Yongqing Liufu), **d** Danish Landrace boars (By Shichong Wang), **e** Hybrid black-spotted white pig (by Yongqing Liufu))

Luchuan pigs may have limited market share due to their small size, slow growth, and low lean meat percentage. However, they possess valuable genetic traits such as high prolificacy, tolerance to coarse feed, high reproductive capacity, early maturation, and easy fattening, making them of significant genetic breeding value. For instance, crossbreeding Luchuan sows with Danish Landrace boars produces hybrid offspring, and further crossing these hybrids with Duroc boars creates three-way crossbred pigs. Likewise, crossbreeding Luchuan sows with Duroc boars yields black pig offspring, and crossing these offspring with Danish Landrace boars results in three-way crossbred pigs (Fig. 5). The hybrid pigs exhibit excellent adaptability, high productivity, and superior meat quality, giving them a competitive edge in the market [53].

In southern China, Luchuan pig is recognized as an ideal hybrid female parent. In the past, Hakka people primarily raised Luchuan pigs to meet their family's protein needs. Currently, due to increasing recognition of the genetic breeding value of Luchuan pigs, many farmers are rearing them with the intention of acquiring more female pigs and subsequently attaining greater economic benefits through hybrid breeding. This practice not only satisfies people's demand for traditional diets but also successfully achieves economic benefits. This farming practice objectively promotes the protection of Luchuan pigs and has a positive significance for safeguarding both the biological and cultural diversity of Luchuan County. Therefore, in the future, while conducting rescue and protection work for traditional knowledge, there should be a focused effort on preserving the genetic resources of local varieties.

The Luchuan pig farming industry is a distinctive sector in Luchuan County. According to statistics, in 2016, the total output value of Luchuan pigs reached 5-billion-yuan, accounting for 70% of the counties' total agricultural output [54]. In recent years, the government has undertaken significant efforts to protect and breed Luchuan pigs. These efforts include establishing a national-level Luchuan pig conservation center, setting up Luchuan pig protection areas in five different towns, providing financial subsidies to Luchuan pig breeders, and promoting the Luchuan pig brand, etc.

Despite these measures, the production and utilization of Luchuan pigs still face several challenges. Firstly, the current sales market for Luchuan pigs mainly relies on local consumption. However, with the impact of low-cost, high-yield pork from Europe and America, it is difficult for Luchuan pigs to fetch competitive prices in the market. Secondly, due to insufficient consumer awareness of Luchuan pigs, fraudulent practices in the pork market led to a significant negative impact on the sales of genuine Luchuan pork. Although the government is working toward building the Luchuan pig brand, there are currently only few Luchuan pig specialty chain stores in Yulin, Guigang, and Nanning in Guangxi, which is insufficient for promoting and disseminating Luchuan pigs. Additionally, epidemics have always posed the most significant obstacle to pig farming [55]. In 2018, African swine fever was introduced into China, resulting in an outbreak. According to disease prevention policies, all deceased pigs, culled pigs, their products, potentially contaminated feed, equipment, and waste materials were subject to safe disposal. Similar to other pigbreed in China [3], during the outbreak of African swine fever, Luchuan pigs suffered significant losses, with the number decreasing from over 1.31 million to 235,000, causing substantial economic damage to breeders [54]. While some interviewees mentioned the effectiveness of strict epidemic prevention measures and feeding “liangcha” (cooling herbal tea) to pigs to reduce the occurrence and spread of African swine fever, most pig breeders lack experience in epidemic prevention and control, leading them to avoid pig farming risks.

Therefore, to promote and protect Luchuan pigs, government agencies, related companies and non-governmental organizations need to take proactive measures, such as (1) increasing the promotion of breeding and farming methods; (2) utilizing the pig farming association platform to educate farmers on preventing and treating common diseases; (3) enhancing research and development in pork processing to expand Luchuan pig meat products in domestic and international markets; (4) conducting effective publicity through museums, promotional events, media, etc., to increase consumer awareness of Luchuan pigs; and (5) attracting investment to establish more specialty chain stores.

### Folk culture and customs related to Luchuan pigs

**Fig. 6:**
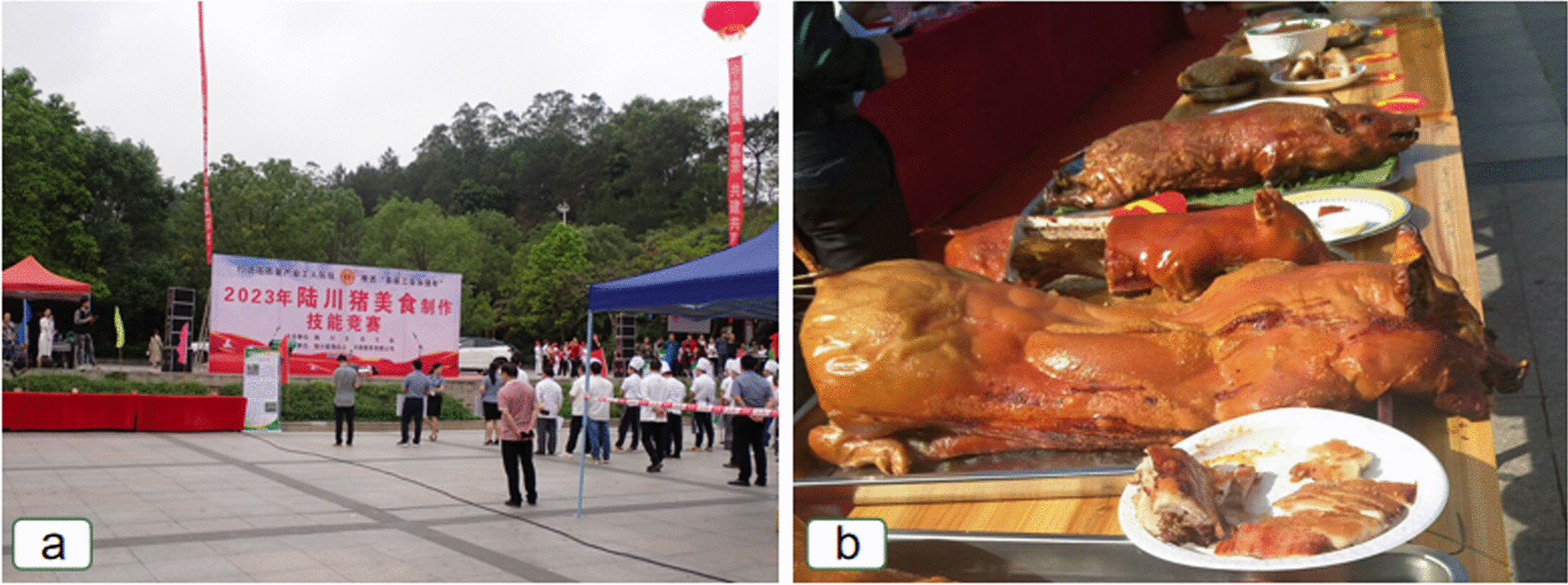
Luchuan Pig Cultural Festival and Luchuan Pig cuisine. (**a** Luchuan Pig cuisine cooking competition (by Yongqing Liufu), **b** Luchuan Pig cuisine (by Weicui Wu ))

Luchuan pigs play a significant role in the local Hakka traditional dietary culture, social rituals, festive celebrations, and customary practices (Fig. 6). Among the Hakka community, Luchuan pigs are regarded as symbols of kindness, good fortune, and blessings. The locals adhere to feeding Luchuan pigs with natural feeds. Luchuan pig meat is highly valued for its delicious taste and nutritional benefits, making it a precious ingredient in both culinary and medicinal practices. Additionally, in the Hakka culture of worship and offerings, pigs are considered the most sincere and meaningful tribute to the deities.

#### Dietary culture

The traditional practice of feeding Luchuan pigs with natural feed is closely related to the lifestyle habits of the Luchuan Hakka people. In the past, the Luchuan Hakka people relished “Lao Fan”, which was a dish made by boiling a large quantity of water with rice in a wok, then scooping out the cooked rice with a ladle. They would mix the leftover rice broth with other plant fodders to feed pigs. Until now, many farmers in rural areas still maintain this traditional method of feed production.

Luchuan pigs belong to the fatty-type breed, characterized by tender, aromatic, crisp, and sweet meat. Locals prepare various pork dishes to entertain guests, creating a rich culture centered around consuming pork. Commonly enjoyed dishes include white-cut pig’s feet, roasted suckling pig, braised pork, cured pork, sausage, and preserved meat. For the Hakka community in Luchuan, the Luchuan pig serves not only as a source of nutritional food but also as an important medicinal resource for preventing and treating illnesses.

Through long-term practical experience, they have developed the concept of “Doctrine of Signature”, using specific parts of the pig to address corresponding health issues. Such as consuming pig's blood to replenish blood, pig's kidneys to nourish the kidneys, and pig's feet to strengthen the feet. Local Hakka people use pig liver, and goji (*Lycium chinense*) leaves together to make soup with the effects of liver clearing and vision improvement. Stewed pig's ears with *Combretum alfredii* are used to treat tinnitus, while a soup made from *Tinospora sinensis* and pig's feet is employed to alleviate rheumatic bone pain and other ailments.

#### Folk festivals and traditional customs

The Luchuan pigs play a significant role in the local Hakka traditional customs. Locally, when a Hakka man gets married, it is customary to present a pig's head and tail to the matchmaker as a gesture of gratitude. Additionally, the Luchuan pig is an essential offering in the worship of deities and ancestors during traditional festivals and important customs among the Hakka people. Particularly during the Qingming Festival and the Double Ninth Festival, affluent Hakka families often use a roasted suckling pig as an offering to their ancestors (Fig. 6b). The Hakka people of Luchuan believe that pigs are easy to raise and bring good fortune. Therefore, if a child is weak and prone to illness, their parents may offer a Luchuan pig or a pig's head to the local earth god, praying for the child to be as healthy and fortunate as a pig.

Amid urbanization and changing lifestyles, many traditional ceremonies are gradually fading from people's view. However, the cultural significance related to pigs remains well-preserved in the Hakka region of Luchuan County. The Luchuan pig not only holds an important place in Hakka culture but also serves as a medium for reflecting the distinct characteristics of the local Hakka people. The Hakka people of Luchuan are known for their kindness, warmth, hospitality, gratitude, and reverence for deities and ancestors. They share the best pork with guests to express hospitality and gratitude and offer pork as the finest tribute to deities and ancestors, conveying their reverence and fond remembrance. These cultural values likely serve as a vital driving force in preserving the local traditional customs and culture. Therefore, while safeguarding the genetic resources of the Luchuan pig, equal emphasis should be placed on the conservation of the associated traditional customs and of Hakka culture.

#### Luchuan pig cultural festival

In recent years, local governments have frequently organized cultural festivals related to the Luchuan pig industry to promote its development. During these cultural festivals, various artists and food enthusiasts utilize the pig as a medium to showcase and celebrate the local Hakka culture, which includes Hakka opera, Hakka folk songs, and Hakka cuisine, among other elements. They employ various forms of expression such as calligraphy, photography, theatrical performances, and culinary demonstrations to promote the Luchuan pig.

Simultaneously, domestic and international pig farming experts, entrepreneurs, and government officials are invited to participate in these cultural festivals. They share and exchange experiences related to Luchuan pig breeding, management, product processing, sales, promotion, and conservation. The rich heritage of Hakka traditional customs and culture infuses cultural elements into the promotion and preservation of the Luchuan pig, further propelling the development of the Luchuan pig industry.

## Conclusion

The Luchuan Hakka people possess rich traditional knowledge and management experience in raising Luchuan pigs. However, with improved living standards, changes in farming practices, and developments in medical technology, many traditional wild feed plants have been replaced by fermented feed and cultivated crops, while traditional Chinese veterinary medicine has been substituted by vaccines and western medicine. The traditional knowledge about using and managing wild feed plants and veterinary plants faces a significant risk of disappearing, and effective measures are needed to preserve it. The ecological farming and under forest grazing models of Luchuan pigs have reduced environmental pollution and potential threats caused by pig farming, promoting the sustainable development of Luchuan pig farming. These models are worthy of promotion. The genetic advantages are an important driving force for the conservation and breeding of Luchuan pigs. Currently, there are still some challenges in raising Luchuan pigs that need to be further improved and protected. As an important local cultural species, efforts should also be made to strengthen the preservation of traditional customs and culture related to Luchuan pigs while protecting their genetic resources.

### Supplementary Information

**Additional file 1:** **Table S2.** The inventory of feed plants for Luchuan pigs.

## Data Availability

All data generated or analyzed during this study are included in this published article.
