# Supplementary material for: Feed plants, ethnoveterinary medicine, and biocultural values: insights on the Luchuan pig from Hakka communities in China
Source: J Ethnobiol Ethnomed. 2023 Sep 15;19:40. doi: 10.1186/s13002-023-00613-4 (PMC10502998; doi:10.1186/s13002-023-00613-4)
Supplement: Supplementary file 1 — Additional file 1: Table S2. The inventory of feed plants for Luchuan pigs. [file 13002_2023_613_MOESM1_ESM.docx]

**Table 2** The inventory of feed plants for Luchuan pigs

| **Specimen No.** | **Family name** | **Scientific name** | **Local name** | **Used part** | **Harvesting season** | **Resource type** | **Life form** | **Integrated score** |
| --- | --- | --- | --- | --- | --- | --- | --- | --- |
| LFYQ23146 | Amaranthaceae | *Beta vulgaris* L. | tóng sháo mài, zhū má cài | leaf | spring, summer, winter | cultivated | herb | 20 |
| LFYQ23157 | Amaranthaceae | *Alternanthera philoxeroides* (Mart.) Griseb. | jiǎ kōng xīn cài | overground part | spring, summer | wild | herb | 16 |
| LFYQ23164 | Amaranthaceae | *Amaranthus viridis* L. | gǒu sè xiàn | whole plant | spring, summer, fall | wild | herb | 16 |
| LFYQ23034 | Amaranthaceae | *Cyathula prostrata* (L.) Blume | xì yàng mǎ biān cǎo , dì dān | whole plant | whole year | wild | herb | 12 |
| LFYQ23019 | Amaranthaceae | *Celosia argentea* L. | qīng xiāng | overground part | spring, summer, fall | wild | herb | 11 |
| LFYQ23144 | Araceae | *Colocasia esculenta* (L.) Schott. | yù tóu miáo , yǎng yù | petiole | whole year | wild | herb | 21 |
| LFYQ23068 | Asteraceae | *Ixeris polycephala* Cass. ex. DC. | mài cài | leaf | spring, winter | cultivated | herb | 21 |
| LFYQ23154 | Asteraceae | *Lactuca sativa* L. | yóu mǎi cài | leaf | whole year | cultivated | herb | 17 |
| LFYQ23159 | Asteraceae | *Lactuca sativa* var. *ramosa* Hort. | shēng cài | leaf | spring, winter | cultivated | herb | 16 |
| LFYQ22018 | Asteraceae | *Emilia sonchifolia* (L.) DC. | yī diǎn hóng | whole plant | whole year | wild | herb | 16 |
| LFYQ23161 | Asteraceae | *Lactuca sativa* var. *angustata* Irish ex Bremer | wō sǔn | leaf | spring, winter | cultivated | herb | 15 |
| LFYQ23111 | Asteraceae | *Erechtites valerianifolius* (Link ex Spreng.) DC. | guò cháo cài | overground part | whole year | wild | herb | 15 |
| LFYQ23048 | Asteraceae | *Gamochaeta pensylvanica* (Willd.) Cabrera | é ài | whole plant | whole year | wild | herb | 15 |
| LFYQ22009 | Asteraceae | *Bidens pilosa* L. | yī bāo zhēn | overground part | whole year | wild | herb | 14 |
| LFYQ23054 | Asteraceae | *Crassocephalum crepidioides* S. Moore | guò cháo cài , gé mìng cài | overground part | spring, summer, fall | wild | herb | 14 |
| LFYQ22172 | Asteraceae | *Eclipta prostrata* (L.) L. | mò cǎo | overground part | whole year | wild | herb | 12 |
| LFYQ23164 | Asteraceae | *Erigeron canadensis* L. | xiǎo péng cǎo | overground part | whole year | wild | herb | 10 |
| LFYQ23150 | Brassicaceae | *Raphanus raphanistrum* subsp. *sativus* (L.) Domin | luó bo | whole plant | winter | cultivated | herb | 18 |
| LFYQ23151 | Brassicaceae | *Brassica rapa* var. *glabra* Regel | huǒ tǒng cài , bái cài | leaf | winter | cultivated | herb | 18 |
| LFYQ23155 | Brassicaceae | *Brassica oleracea* var. *botrytis* L. | yē zǐ cài | overground part | spring, winter | cultivated | herb | 17 |
| LFYQ23156 | Brassicaceae | *Brassica oleracea* L. | bāo cài | overground part | spring, winter | cultivated | herb | 17 |
| LFYQ23160 | Caricaceae | *Carica papaya* L. | mù dōng guā | fruit | fall | cultivated | tree | 15 |
| LFYQ23020 | Caryophyllaceae | *Stellaria aquatica* Scop. | má sī cǎo, é cháng cài | overground part | whole year | wild | herb | 12 |
| LFYQ23151 | Commelinaceae | *Commelina diffusa* Burm.f. | ròu cǎo , zhú gāo cǎo | whole plant | whole year | wild | herb | 18 |
| LFYQ23142 | Convolvulaceae | *Ipomoea batatas*  (L.) Lam. | hóng shǔ téng , hóng shǔ téng , fān shǔ | whole plant | whole year | cultivated | herb | 24 |
| LFYQ23158 | Convolvulaceae | *Ipomoea aquatica* Forssk. | kōng xīn cài | overground part | summer | cultivated | herb | 16 |
| LFYQ23100 | Costaceae | *Hellenia speciosa* (J.Koenig) Govaerts | fú shǒu gùn | overground part | spring, summer, fall | wild | herb | 11 |
| LFYQ23148 | Cucurbitaceae | *Cucurbita moschata* Duchesne | nán guā | fruit | summer, fall | cultivated | herb | 19 |
| LFYQ23153 | Cucurbitaceae | *Benincasa hispida* Cogn. | dōng guā | fruit | summer, fall | cultivated | herb | 18 |
| LFYQ23145 | Euphorbiaceae | *Manihot esculenta* var. *Pohlii* Cif. | mù shǔ | tuber | fall, winter | cultivated | shrub | 21 |
| LFYQ23143 | Fabaceae | *Glycine max* (L.) Merr. | dòu pò | seed | fall | cultivated | herb | 23 |
| LFYQ23147 | Fabaceae | *Arachis hypogaea* L. | huā shēng fū | seed coat | summer | cultivated | herb | 20 |
| LFYQ23149 | Moraceae | *Broussonetia papyrifera* (L.) Vent. | gòu shù | tender leaf | whole year | wild | tree | 19 |
| LFYQ23148 | Onagraceae | *Ludwigia adscendens* (L.) H. Hara | guò táng shé | whole plant | whole year | wild | herb | 19 |
| LFYQ23021 | Onagraceae | *Ludwigia hyssopifolia* (G. Don) Exell. | / | overground part | spring, winter | wild | herb | 12 |
| LFYQ22784 | Poaceae | *Zea mays* L. | yù mǐ | seed, stem | fall | cultivated | herb | 24 |
| LFYQ22808 | Poaceae | *Oryza sativa* L. | zhōu , mǐ kāng , xǐ mǐ shuǐ | seed, husk | summer, fall | cultivated | herb | 24 |
| LFYQ23052 | Poaceae | *Pennisetum purpureum* Schumach. | tián xiàng cǎo , jiǎ gān zhè | overground part | whole year | cultivated or wild | herb | 24 |
| LFYQ22806 | Poaceae | *Triticum aestivum* subsp. *spelta* (L.) Thell. | mài pí | seed coat | / | introduced | herb | 23 |
| LFYQ23152 | Poaceae | *Cenchrus flaccidus* (Griseb.) Morrone | huáng zhú cǎo | overground part | whole year | wild | herb | 18 |
| LFYQ23018 | Poaceae | *Eleusine indica* Gaertn. | niú jīn cǎo | overground part | whole year | wild | herb | 13 |
| LFYQ23050 | Polygonaceae | *Rumex crispus* L. | jiǎ mài cài | overground part | spring, summer, fall | wild | herb | 16 |
| LFYQ23015 | [Polygonaceae](http://www.iplant.cn/info/Polygonaceae) | *Persicaria maculosa* Gray | xiǎo là liǎo | whole plant | whole year | wild | herb | 15 |
| LFYQ23006 | Polygonaceae | *Persicaria lapathifolia* (L.) Delarbre | jiǎ là liǎo | overground part | whole year | wild | herb | 15 |
| LFYQ23002 | Polygonaceae | *Polygonum plebeium* R. Br. | wū yíng yì , páng xiè yǎn | whole plant | whole year | wild | herb | 13 |
| LFYQ23152 | [Pontederiaceae](http://www.iplant.cn/info/Pontederiaceae) | *Pontederia crassipes* Mart. | shuǐ piāo , fú shuǐ lián | overground part | spring, summer | wild | herb | 18 |
| LFYQ23066 | Portulacaceae | *Portulaca oleracea* L. | mǎ chǐ xiàn | whole plant | spring, summer, fall | wild | herb | 16 |
| LFYQ23163 | Sapindaceae | *Litchi chinensis* Sonn. | lì zhī | leaf | whole year | cultivated | tree | 10 |
| LFYQ23013 | Saururaceae | *Houttuynia cordata* Thunb. | yú xīng cǎo | whole plant | whole year | wild | herb | 16 |
| LFYQ23157 | Solanaceae | *Solanum americanum* Mill. | bái huā cài | stem and leaf | spring, summer, fall | wild | herb | 17 |
| LFYQ23162 | Solanaceae | *Physalis angulata* L. | dēng lóng cài | overground part | whole year | wild | herb | 12 |
